# Weak tides during Cryogenian glaciations

**DOI:** 10.1038/s41467-020-20008-3

**Published:** 2020-12-04

**Authors:** J. A. Mattias Green, Hannah S. Davies, Joao C. Duarte, Jessica R. Creveling, Christopher Scotese

**Affiliations:** 1 School of Ocean Sciences, Bangor University, Menai Bridge, UK; 2 Instituto Dom Luiz (IDL), Faculdade de Ciências, Universidade de Lisboa, Lisboa, Portugal; 3 Departamento de Geologia, Faculdade de Ciências, Universidade de Lisboa, Lisboa, Portugal; 4 School of Earth, Atmosphere and Environment, Monash University, Melbourne, VIC Australia; 5 College of Earth, Ocean, and Atmospheric Sciences, Oregon State University, Corvallis, OR USA; 6 Earth and Planetary Sciences, Northwestern University, Evanston, IL USA

**Keywords:** Ocean sciences, Palaeoceanography, Physical oceanography

## Abstract

The severe “Snowball Earth” glaciations proposed to have existed during the Cryogenian period (720 to 635 million years ago) coincided with the breakup of one supercontinent and assembly of another. Whereas the presence of extensive continental ice sheets predicts a tidally energetic Snowball ocean due to the reduced ocean depth, the supercontinent palaeogeography predicts weak tides because the surrounding ocean is too large to host tidal resonances. Here we show, using an established numerical global tidal model and paleogeographic reconstructions, that the Cryogenian ocean hosted diminished tidal amplitudes and associated energy dissipation rates, reaching 10–50% of today’s rates, during the Snowball glaciations. We argue that the near-absence of Cryogenian tidal processes may have been one contributor to the prolonged glaciations if these were near-global. These results also constrain lunar distance and orbital evolution throughout the Cryogenian, and highlight that simulations of past oceans should include explicit tidally driven mixing processes.

## Abstract (web summary)

How and why the ‘Snowball Earth’ occurred during the Cryogenian period is debated. Here, the authors show that the cryogenian ocean hosted diminished tidal amplitudes and associated energy dissipation rates, reaching 10-50% of today’s rates thus perhaps contributing to prolonged glaciations.

## Introduction

It has been suggested that the Earth experienced near-global severe glaciations during the Cryogenian period (720–635 Ma), events which earned the nickname “Snowball Earth”^1,2^. The earliest Cryogenian glaciation proposed, the Sturtian from 717–660 Ma^1–3^, and the younger Marinoan glaciation, from 650–635 Ma^1,3^, had continental ice advance down to very low latitudes^4^, possibly leaving an open equatorial ocean (the latter known as a “Slushball Earth”^5^). A Snowball state is climatologically stable, with the predicted duration of long-lived glaciation commensurate with the time for volcanic outgassing of greenhouse gases to reach a threshold for deglaciation^1,6–8^, leading to abrupt warming and hothouse conditions after the glaciations^7,9^. Here we propose that a second factor, ocean tides, influenced the duration of Cryogenian Snowball glaciations. Coupled ice flow–ocean circulation models^10,11^ suggest that there was only a single vigorous meridional overturning circulation cell, and hence stratification, near the equator in the Snowball ocean. The rest of the ocean was most likely vertically mixed or only very weakly stratified because of strong convective overturns from geothermal heating^11,12^. If tidal dissipation, i.e., the loss of tidal energy due to boundary friction and tidal conversion (the generation of internal tidal waves), was then added to the background flow, the stratification could break down further^13^. This scenario predicts negligible tidal conversion (i.e., the generation of an internal tide), and tidal dissipation would be limited to the frictional boundary layer near the sea floor and underneath the ice. It has been suggested that tides in the vicinity of the Laurentide ice sheet during the last deglaciation probably contributed to its rapid collapse^14^. The melt rate in cavities under the ice shelf in present day Antarctica is largely controlled by tidally driven mixing, because mixing stirs the cold and fresh meltwater under the ice down into the water column, thus allowing saltier and warmer water to be brought into contact with the ice^15^. Breaking down the saline stratification in the ice-ocean boundary layer is a key process that will happen even if the rest of the ocean is only weakly stratified. Thus, weak tides would reduce under-ice mixing rates, which could prolong the duration of a Snowball glaciation, with far-reaching consequences for the Earth system.

Tides are known to fluctuate on geological time scales^16,17^ due to changes in the basin geometries induced by the motion of the Earth’s tectonic plates^18,19^. The main mechanism for amplification of the tides is tidal resonance, which occurs when the size of a basin is equal to half a wave length of the tidal wave^20,21^. Because of movements of the tectonic plates, we can therefore expect the tides to change on scales of millions of years. Also, because the wavelength is set by the tidal period (here taken to be 10.98 h throughout the period under investigation^22,23^—see our methods for more details) and the speed of the wave, which in turn is set by the water depth, large-scale variations in depth due to the appearance of ice can also move a basin towards, or away from, resonance on scales shorter than those of tectonic motions.

Here, we aim to quantify Cryogenian tidal energetics by simulating the evolution of the global tides using 20 recent paleo-geographic reconstructions covering 750–500 Ma^18^ in a numerical tidal model^17^ (see Methods for details and sensitivity simulations). We discuss how Cryogenian tidal amplitude and dissipation was affected by and could have contributed to the onset and termination of Snowball glaciation, and wider implications of the tidal results. The investigation covers the late Neoproterozoic, including the Cryogenian, and spans 750–600 Ma. We model a Sturtian and Marinoan glaciation duration from 715–660 Ma and 650–635 Ma, respectively.

## Results

### Tidal amplitudes

**Fig. 1:**
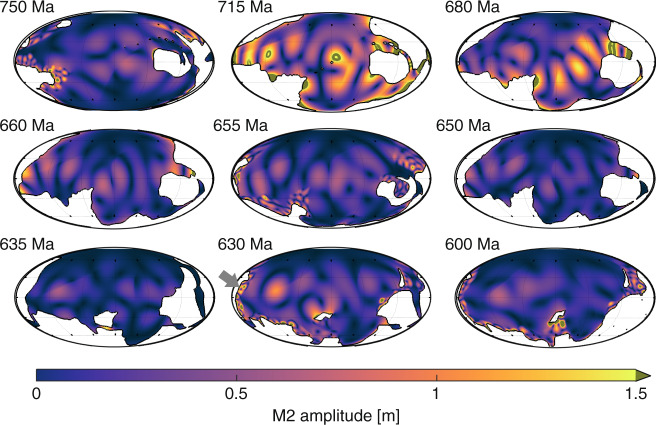
Simulated M2 tidal amplitudes in metres for the time slices representing 750, 715, 680, 660, 655, 650, 635, 630 and 600 Ma (see labels in the top left hand corner of each panel; note that all global maps are plotted on a Mollweide projection). Note that the colour scale saturates at 1.5 m, in a grey-green colour, for clarity. The grey arrow at 630 Ma point to the coastline where the Elatina formation^31^ is now located. The formation gives a tidal proxy showing a range consistent with the one presented from the model.

**Fig. 2:**
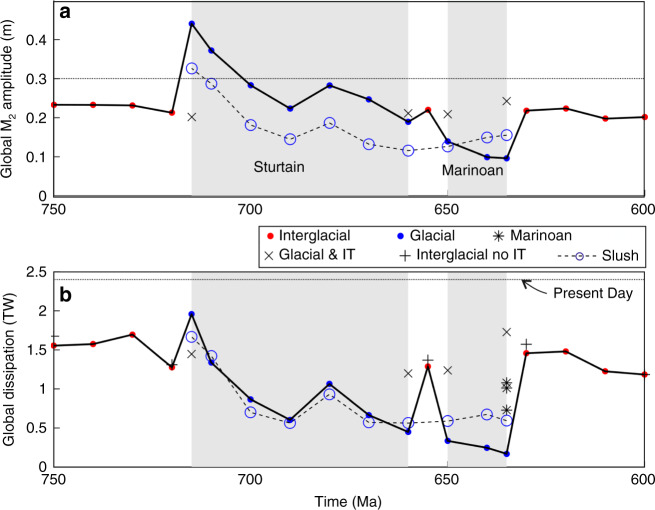
The evolution of globally averaged tidal parameters during the Cryogenian. Globally averaged M_2_ amplitudes (**a**) and integrated dissipation rates (**b**) throughout the period under consideration. The black solid line is the result from the set of simulations with conversion and no sea level change during interglacial periods (red dots), and a 500 m lowstand, no conversion, and increased bed friction during the glacial periods (blue dots). The x-symbols mark sensitivity simulations at the onset and end of glacial periods, in which non-glacial conditions were used, and the plus signs (+) mark simulations during interglacial periods without tidal conversion. The blue dashed line with circle markers shows the results for the Slushball with an ice-free band within 10^o^ from the equator. The horizontal black dotted lines mark present day values and the blue shaded areas mark the spans of the two glaciations.

The numerical simulations predict global mean M2 tidal amplitudes of ~0.2 m prior to the onset of the Sturtian glaciation (Figs. 1 and 2a, and Supplementary Fig. 1; note that the tidal range is twice the amplitude). At 715 Ma, model glacial tidal amplitudes rapidly increase to 0.44 m, higher than present-day tidal amplitudes (Fig. 2), due to sea-level fall below the continental shelf. This allows a tidal resonance to develop, much like the enhanced resonance during the Last Glacial Maximum^21,24^. The tidal amplitude in the simulations then decreases during the next 25 Ma due to a tectonic configuration that was unable to host a large tide because the basins were too large to be near resonance for the semidiurnal tide^21,25–27^. The model suggests that at 680 Ma, the tide became more energetic again because the tectonic emergence of land over the South Pole and a convergence of the main continental landmasses in the southern hemisphere changed the geometry of the large superocean basin to a size that was closer to that required for tidal resonance. Another decrease in tidal amplitude would have occurred through ~660 Ma because the continental configuration would only have allowed for small tidal amplitudes. The tide at 655 Ma, however, is slightly elevated in the model because the continental configuration allowed for a large tide between the glaciations. The onset of the model Marinoan glaciation at 650 Ma again reduced the tidal amplitude, resulting in the most tidally quiescent period in all deep-time simulations to date^17,28^. Finally, deglaciation tidal amplitudes recover in the model to about 0.2 m between 630 and 600 Ma. The results highlight a tide-ice feedback in which the tidal dissipation response for the Sturtian glaciation is similar to that during the Pleistocene glaciations, where the sea-level lowstand enhanced dissipation due to ocean resonances^21,24,29^. In contrast, during the Marinoan glaciation, the ice weakened the tides by an enhanced friction and changes in water depth that prohibited resonances to develop.

The modelled amplitudes are on average around 0.2 m throughout the Cryogenian, or about 2/3 of present-day values. This may not amount to a very large difference, but it is generally the tidal dissipation rates that are of importance to the wider Earth system, including driving under ice melting and large-scale ocean circulation patterns.

### Tidal dissipation

The tidal dissipation rates computed from the simulations (Fig. 2b) are consistently below modern values. The estimated peak tidal dissipation rate at 715 Ma is 2TW, or 80% of today’s rate (dashed line in Fig. 2b), while the minimum tidal dissipation rate in the Marinoan simulation is only about 10% of modern values^30^. This supports the hypothesis put forward here that Cryogenian glaciations damped global tides and tidally driven processes. A key feature, however, is the very sharp rise in the dissipation rates at the end of the Marinoan; over 5 Myr the tidal dissipation rate increases from 0.2 to 1.4 TW. Deglacial ice melt thus had important effects on the tides as ocean circulation and tidally driven mixing recovered. As the reconstructed continental configuration changed minimally between 635 and 630 Ma, the change in tidal amplitude and dissipation in the model arises from the parametrization of the ice sheets (i.e., the lowstand in sea level and changes in friction and tidal conversion discussed above). Glacial suppression of the tides is supported by simulations using interglacial conditions (i.e., sea level high-stand, reduced friction, and tidal conversion) for the beginning and end points for each glaciation, which show results in line with the nearby interglacial time slices (x symbols in Fig. 2b). In contrast, sensitivity simulations for interglacial time slices without tidal conversion (+ symbols in Fig. 2b) show only a minor change in the tides, further supporting the robustness of these results. Also, the simulations from 630 Ma show localised large amplitudes of over 2 m along the coastline of what is today south Australia (see the grey arrow in Fig. 1 for the location). This is the location of the tidally influenced Elatina formation^31^, and our amplitudes match those described from the site.

In our Snowball simulations we assume that the entire ocean was ice covered. As mentioned above, Earth may instead have been in a Slushball state, where the equatorial ocean was ice free^32^. Consequently, we simulated the Slushball for the glaciated time slices by allowing a full water depth within 10^o^ from the equator and having a weak stratification throughout the ocean (see Methods for details and Supplementary Figs. 2 and 3). The average M2 amplitude and associated integrated dissipation rates are again shown in (Fig. 2 as blue circles on a dashed line). Interestingly, these simulations show weaker tidal amplitudes, except for the end of the Marinoan, and the tidal dissipation rates are below those of the snowball for most time slices. The reason for this response is that when tidal conversion is re-introduced in the deep ocean, the amplitudes are reduced, especially in the shallow shelf seas present, and thus there is less dissipation of energy in total. This is due to the non-linear interplay between friction and conversion, as seen in simulations for the Last Glacial Maximum (21ka)^29,33^. These results further show that the Cryogenian tides were weak, regardless of how severe the glaciations were, and we argue that this supports our idea that weak tides were a key process in the Cryogenian ocean.

*Details of the Marinoan deglaciation*: The duration of deglaciation predicted by the Snowball hypothesis^1^ is shorter than the 5 Myr model resolution adopted here. The increase in tidal amplitude after the glaciations, at both 655 Ma and 630 Ma, raises the question of how fast tides respond to deglaciation? To address this question, we used the three Marinoan deglacial bathymetries^34^ with higher temporal resolution, covering 0 kyr, 2 kyr and 10 kyr from the initiation of the deglaciation (these simulations were done for a Snowball state only, as the tides were weakest in this state for this period, see; Fig. 2). The results show a rapid increase in tidal dissipation, from 0.7 TW at 0 kyr to 1.1 TW at 10 kyr, consistent with the deglacial signal between 635 and 630 Ma (note that the simulations, shown as black asterisks in Fig. 2b, appear at the same point on the x-axis because of the short time span relative to the full simulation). Thus, the deglacial rise in the tidal amplitude and dissipation would have occurred over millennia, rather than millions of years. Notably, the difference in tidal dissipation between the 635 Ma and the 0 kyr simulation, a factor 3.5 (from 0.2 to 0.7 TW), provides an estimate of uncertainty in the simulations. The 635 Ma simulation likely underestimates tidal dissipation due to the uniform 500 m sea level decrease, whereas the 0 kyr simulation includes a spatially varying sea level fingerprint. Furthermore, by excluding deep ocean bathymetry in the Marinoan reconstructions we overestimate tidal dissipation rates by up to a factor 2^17,24^. The key conclusion of this investigation, however, is not in the exact amplitude or dissipation rate—they both require knowledge of the Late Neoproterozoic Earth system beyond that preserved in the rock record––but rather the robust result that Snowball glaciation led to generally very small tidal amplitudes, and that rapid deglaciation allowed the tides to recover.

## Discussion

There is uncertainty in the paleogeographic reconstructions for the Cryogenian^18,34^. Our tidal results are representative of scenarios of global glaciation of a specific ice/ocean volume, and may differ substantially under alternative scenarios of ice volume and distribution^35^. The sea-level changes we used here are based on the commonly cited assumptions of glacial volume and deglacial timescale^1^. Our globally integrated results are robust and the sensitivity simulation only change the globally integrated tidal dissipation rates by less than 10%. This holds for our Slushball simulations as well. These have an ice-free ocean around the Equator, and a weak stratification allowing for open ocean energy losses through tidal conversion (blue dashed lines in Fig. 2). The largest difference in the tides is seen at the onset and end of the glaciations, in simulations both with and without the ice parameterisation (i.e., double friction, lower sea-level, and no conversion—see Methods for details; Fig. 2b). The tidal signal that then emerges can be explained by how the differences in glacial reconstructions would affect tides, and it shows the effect of the deglaciation on the tides.

These results highlight a connection between oceanography (tides) and palaeogeography (ultimately set by tectonics) in the climatic stability of a Snowball Earth. Quiescent tides during Snowball glaciations could have contributed to climate stability, because tidally driven processes, acting to melt ice by destabilising the freshwater stratification near the ice and allowing warmer water into contact with the ice, were severely muted for millions of years (or longer for the Sturtian). Tides are of course not the only process influencing the ice-sheets – if they were the main controller the Marinoan should have lasted longer than the Sturtian. However, the tides are a potential mechanism for destabilization of the ice once it starts to collapse. We also show that tides and tectonics are not independent on geological time scales: for a large fraction of the late Neoproterozoic, including the Cryogenian, Earth was in a supercontinent state. This led to weak late Neoproterozoic tides because of a lack of resonant ocean basins, except locally during a few time slices. The Cryogenian is the most quiescent period of the 1 Gyr of Earth’s tides simulated to date^17,28^. The resulting low tidal energy and tidal mixing would have had consequences for other components of the Earth system, including ocean circulation patterns and vertical fluxes of mass, salt, heat, and tracers, and for the evolution and dispersion of Neoproterozoic life. Detailed investigations of these consequences are left for future studies. The results also suggest that conceptual models of Cryogenian tides on Earth^36^, may not necessarily provide converging results when compared to explicitly simulated tides with realistic paleo-geographies. We confirm the existence of the supertidal cycle, a long-term cycle of tidal strength, which is tied to the supercontinent assembly and dispersal^28^. This has further implications for the Earth system, because tidal drag induces lunar recession^17^, and the current recession rate is too large to support the old Moon age model^37^. The tidal dissipation rates must therefore have been weaker than at present for prolonged periods of Earth’s history, and our results provide support for this being the case.

## Methods

Late Neoproterozoic tides were simulated using a dedicated numerical tidal model^17,21,24,28,29,38^ that parameterizes energy losses due to both friction at the sea floor and tidal conversion. The latter includes the buoyancy frequency as a measure of vertical stratification, which is uncertain for ancient oceans. Consequently, we adopted values based on observed present day values for non-glaciated time slices^39^ and a non-stratified ocean for all time slices representing Snowball states^11^. The effect of friction in the glaciated time slices was enhanced with respect to the non-glaciated time slices to represent the presence of thick ice covering the ocean (see below for details). We adopt an Earth-moon orbital configuration consistent with the Late Neoproterozoic, including a 21.9 h solar day^31^, a 10.98 h lunar period, and a lunar forcing 15% larger than the modern. Neoproterozoic paleobathymetries were created from recent reconstructions^18^ and interpolated using the GPlates software^40,41^ to obtain bathymetries every 10 Myr from 750–600 Ma interval, with three extra slices produced for 715 Ma (the onset of the Sturtian), 655 Ma (the interglacial), and 635 Ma (the end of the Marinoan). In the non-glacial time slices, ocean volume was set to the same as for present day, whereas glaciated time slices included a lowstand of 500 m. We also used three slices from Creveling and Mitrovica^34^ representing the termination of the Marinoan glaciation (0 kyr), and 2 kyr and 10 kyr into the deglaciation, respectively^34^.

### Tidal modelling

The Oregon State University Tidal Inversion Software (OTIS) has been used extensively to simulate deep-time, present day, and future tides^17,21,24,28,29,38^, and it has been benchmarked against other forward tidal models^42^. It provides a numerical solution to the linearised shallow water equations, with the non-linear advection and horizontal diffusion excluded without a loss in accuracy^24^:

1 $$\frac{{\partial {\mathbf{U}}}}{{\partial t}} + f \times {\mathbf{U}} = gH\nabla ( {\eta - \eta _{{\mathrm{SAL}}} - \eta _{{\mathrm{EQ}}}} ) - {\mathbf{F}}$$

2 $$\frac{{\partial \eta }}{{\partial t}} - \nabla \cdot {\mathbf{U}} = 0$$

Here, **U** = **u***H* is the tidal volume transport (**u** is the horizontal velocity vector and *H* is the water depth), *f* is the Coriolis parameter, *g* is acceleration due to gravity, *η* is the sea-surface elevation, *η*_SAL_ is the self-attraction and loading elevation, *η*_EQ_ is the elevation of the equilibrium tide, and **F** the tidal energy dissipation term. This consists of two parts, **F** = **F**_B_ + **F**_W_, where **F**_B_ parameterizes bed friction and **F**_W_ represents energy losses due to tidal conversion, i.e., due to the generation of a baroclinic tide. Bed friction is parameterised through the standard quadratic law, **F**_**B**_ = *C*_*D*_**u** | **u** | , where *C*_*D*_ = 0.003 is a dimensionless drag coefficient. In the glaciated time slices, *C*_*d*_ = 0.006 was used to represent the effect of the ice covering the ocean as it effectively sets up a second boundary layer. The tidal conversion term is given by **F**_W_ = C**U**, and the conversion coefficient, C, was given by^39,43,44^

3 $$C\left( {x,y} \right) = \gamma \frac{{N_H\bar N}}{{8\pi \omega }}\left( {\nabla H} \right)^2$$

Here, *γ* = 100 represents a dimensionless scaling factor representing unresolved bathymetric roughness, *N*_*H*_ is the buoyancy frequency at the seabed, $\bar N$ represents the vertical average of the buoyancy frequency, and *ω* is the frequency of the tide. The buoyancy frequency, *N*, is given by *N*^2^ = −*g/ρ ∂ρ/∂z*, where *ρ* is the density. The distribution of *N* is based on a statistical fit to observed present day values^39^, or *N(x,y) = 0.00524exp(−z/1300)*, where *z* is the vertical coordinate, and the constants 0.00524 and 1300 have units of s^−1^ and m, respectively. We do not change these values of N in our simulations, but rather test sensitivity by modifying γ (because details of N is largely unknown for the period). The exception is the Snowball oceans, which were only weakly stratified^11^, and the conversion was then switched off by setting *γ* = 0. To test the robustness of the parameterisation, sensitivity simulations were done for several time slices. For those at the beginning and end of each glaciation (i.e., 715, 660, 650, and 635 Ma), we did further simulations with *γ* = 100, and for select non-glacial states (600, 630, 655, 720, and 750 Ma) sensitivity tests were done with *γ* = 200 or *γ* = 0, representing a strongly stratified or unstratified ocean, respectively.

Our Slushball state was simulated by allowing for an open ocean within 10^o^ of the equator. This was implemented by an exponential change in water depth over 1^o^ in latitude from the 500 m lowstand to the ice-free ocean and then doubling the bed friction under the ice only. The Slushball ocean was likely weakly stratified, so we re-introduced a weak tidal conversion by setting γ = 50 in Eq. (3).

### Bathymetry

**Fig. 3:**
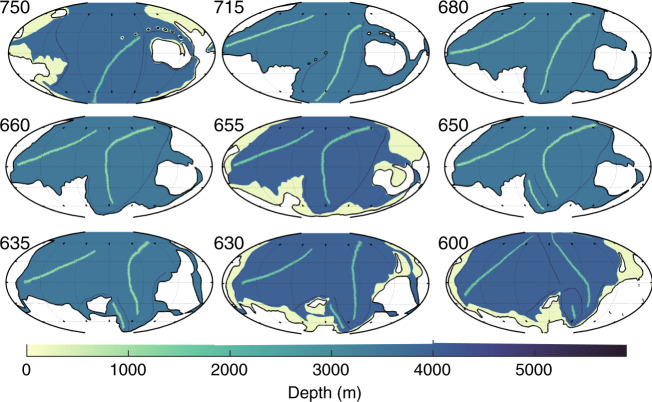
The ocean bathymetry for nine key time slices. Shown is the ocean bathymetry, with depths in colour land white. Note the age for each time slice marked by the label at top left corner, and the black contour marking the coastline and the lack of shelf seas during the glaciations (715–660 Ma and 650–635 Ma). The green lines in the deep ocean mark the peaks of the oceanic ridges and the dark grey lines show trenches.

The paleo-bathymetries for the Snowball simulations were created by digitising reconstructions of the late Proterozoic^18^, using GPlates^40,41^. The original reconstructions covered every 50 Ma between 600–750 Ma, so to improve the temporal resolution, the information was interpolated linearly between these slices to obtain bathymetries every 10 Ma in our 600–750 Ma interval. Furthermore, three extra slices were produced for 635 Ma (end of the Marinoan), 655 Ma (interglacial), and 715 Ma (onset of the Sturtian). The resulting 19 images were then translated to ocean bathymetries by setting continental shelf seas to 200 m depth, and subduction zones to 5900 m. Mid-oceanic ridges were 2500 m deep at the crest, and sloped linearly into the abyss over 5° in width. The abyssal plains were set to a depth that conserved present day ocean volume once all the other bathymetric features were set. There is obviously uncertainty in the Cryogenian sea level, although it is clear that it must have been low during the glaciations; Creveling & Mitrovica^34^ suggest a lowstand of up to 1500 m below interglacial levels at some locations, and a mean sea level 500–800 m below interglacial levels. Consequently, we reduced the depth in our glaciated time slices by 500 m to represent the lowstand (simulations with 800 m lowstand do not change the qualitative results). The grids used in the simulation for selected time slices are shown in Fig. 3.

When we mention glacial conditions, we thus refer to a situation with a doubled bed friction (to represent the ice boundary layer), g = 0 (to represent an unstratified ocean), and sea-level lowered by 500 m, and non-glacial simulation uses the default parameters discussed above.

**Fig. 4:**
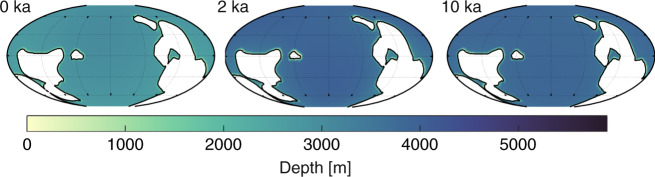
The ocean bathymetry during the Marinoan deglaciation. From left to right are the bathymetry for the 0 kyr, 2 kyr, and 10 kyr time slices, with times measured from onset of the Marinoan deglaciation^34^.

Furthermore, simulations of the relative sea level changes during the Marinoan deglaciation around 635 Ma for three slices were also used^34^. These represent the termination of the glaciation (i.e., 0 kyr after our 635 Ma slice), and then 2 kyr and 10 kyr into the deglaciation (this last slice represents the end of the deglaciation; see Fig. 4). We used these slices for a set of sensitivity simulations and refer to them as 0 kyr, 2 kyr, and 10 kyr in the following, or as “the Marinoan” when discussed as a group. This gives us a unique opportunity to add 3 simulations at higher temporal resolution to further evaluate the influence of the glaciations on the tides.

### Simulations and computations

The Earth-moon system’s orbital configuration was different during the Cryogenian, and here we used a 21.9 h solar day^45^, a 10.98 h lunar period, and lunar forcing 15% larger than at present day^17,23^. Simulations were done for all 19 time slices with a range of parameter choices to ensure the results were robust. The effect of the ice-sheet was parameterised in the Snowball time slices by neglecting conversion (i.e., with *γ* = 0 in Eq. (3); the Snowball state was most likely unstratified^10^), a doubled drag coefficient (i.e., *C*_*d*_ = 0.006) and with a 500 m uniform lowstand in sea level to represent the effect of the ice. Note that floating ice does not impose a rigid lid for the tide because the ice moves with sea surface. Landfast ice without fractures may act as a lid in smaller regions not resolved here. The enhanced drag coefficient is justified by the rough underside of the ice, which leads to effective energy losses in ice covered areas^46^. This may lead to tidally driven residual currents as well, and these may be important because of the quiescent ocean. Analysing them is left for future studies. The time slices at onset and termination of the glaciations (i.e., 715, 660, 650, and 635 Ma) were also simulated without the lowstand and with conversion to represent non-glaciated states. Furthermore, the non-glaciated time slices at 750, 720, 655, 630 and 600 were used to test the robustness of the conversion parameterisation and rerun with *γ* = 200 (i.e., representing a very strong, doubled, vertical stratification). A further set of sensitivity simulations for these slices had further doubling and halving of the drag coefficient, *C*_*d*_, and/or the conversion scaling factor, γ. As in other deep-time studies^17,43^, the sensitivity simulations (not shown) only led to limited changes in global dissipation rates, and we conclude that the results presented here are robust. The Slushball simulations are described above.

Simulations for all of our time slices were done at 1/4^o^ horizontal resolution in both latitude and longitude, achieved through linear interpolation from the original data described above. Each simulation covered 14 days, of which 5 days were used for harmonic analysis of the tide. Simulations were done for the two dominating constituents, M2 (principle lunar) and K1 (luni-solar declination), although focus in the following is on M2. The model outputs amplitudes and phases for the surface elevation and transport vector for each simulated tidal constituent.

The model output was used to compute tidal dissipation rates, *D*, as the difference between the time average of the work done by the tide generating force (**W**) and the divergence of the horizontal energy flux (**P**)^47^:

4 $$D = {\mathrm{W}} - \nabla \cdot {\mathbf{P}}$$

where W and **P** are given by

5 $$W = g\rho \langle {\mathbf{U}} \cdot \nabla (\eta _{EQ} + \eta SAL)\rangle$$

and

6 $${\mathbf{P}} = \,g\rho \langle {\mathbf{U}}\eta \rangle$$

In Eqs. (5) and (6) the angular brackets mark time-averages over a tidal period.

### Present day validation

The core model set-up used here is the same as in other deep-time tidal simulations^17,48^, and it is briefly described here. A present day control simulation^17^ gives a root-mean-square error of about 11 cm for the M2 tidal amplitudes when compared to the data in TPXO8 (http://www.tpxo.net). A simulation with a degenerated present day bathymetry, with less resolution to represent reconstructed bathymetry, produced an error of about 20 cm^43^. It also produces an M2 dissipation rate that is 75% higher than in the present day simulation because of a lack of deep-ocean bathymetry^17^. It is thus highly likely that our simulations overestimate the Cryogenian tidal dissipation rates, especially in the Marinoan simulations.

## Supplementary information

Supplementary Information

## Data Availability

The manuscript data are available from the Open Science Framework (https://osf.io/wnyhr/?view_only=bae47b065db24d97b79d106536a59549).
