# Supplementary material for: Weak tides during Cryogenian glaciations
Source: Nat Commun. 2020 Dec 4;11:6227. doi: 10.1038/s41467-020-20008-3 (PMC7718895; doi:10.1038/s41467-020-20008-3)
Supplement: Supplementary file 1 — Supplementary Information [file 41467_2020_20008_MOESM1_ESM.pdf]

Weak tides during Cryogenian glaciations

J. A. Mattias Green, Hannah S. Davies, Joao C. Duarte, Jessica R. Creveling, Christopher Scotese

Supplementary Material

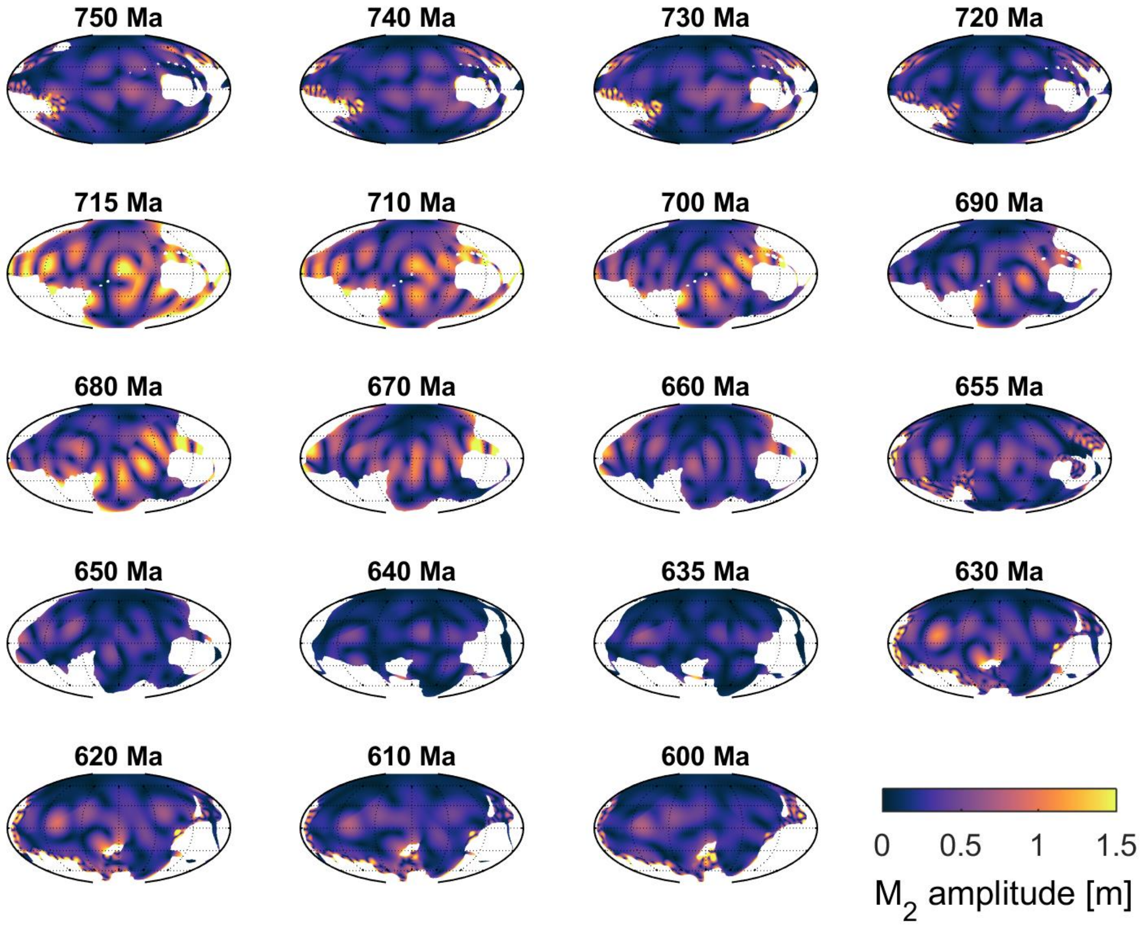

Supplementary Figure 1: The modelled M2 tidal amplitudes for all 19 slices discussed in the paper. The age is provided above each panel. Note that the simulations for 635-650 Ma and 660-715 Ma are glaciated with Snowball conditions, whereas the other slices are ice free.

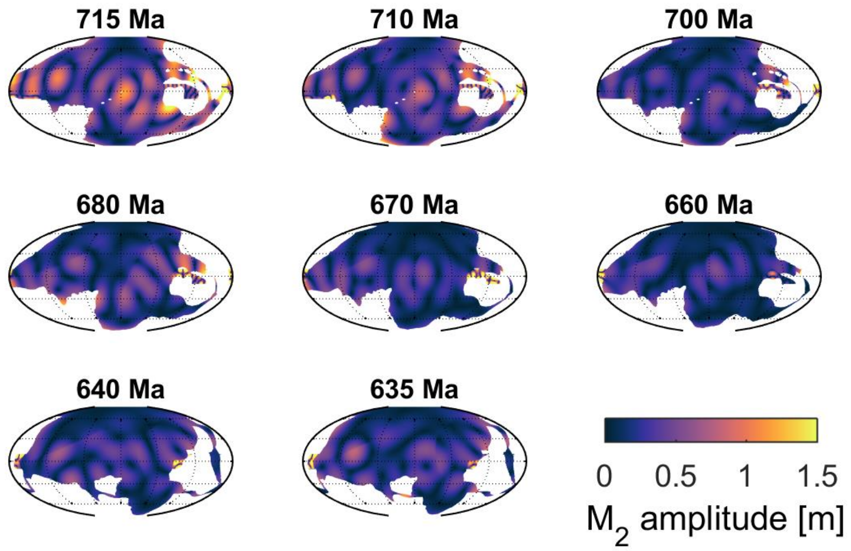

Supplementary Figure2: As in Supplementary Figure 1, but showing the glaciated Slushball simulations with tidal conversion based on present day.

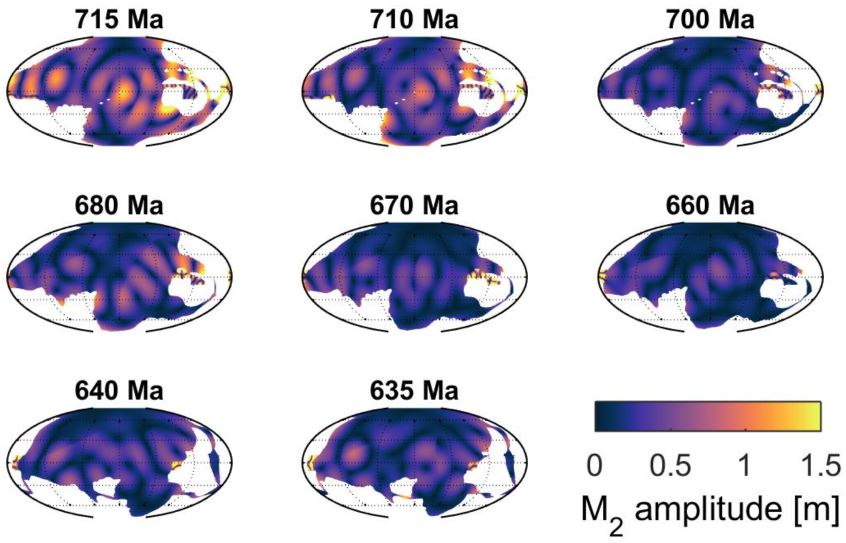

Supplementary Figure 3: As in Supplementary Figure 2, but showing the glaciated Slushball simulations with tidal conversion reduced to half of that in Supplementary Figure 2.
